# Barriers and Facilitators of Oral PrEP Uptake Against HIV Among Selected Priority Populations: A Global Perspective

**DOI:** 10.1007/s11904-026-00783-2

**Published:** 2026-03-30

**Authors:** Ifeanyi Ngwoke, Adepeju Mary Adedire, Nengak Precious Danladi, Chinemerem Cynthia Eze, Oluwasogo Kehinde Dada, Olalekan John Okesanya

**Affiliations:** 1 Royal Dick(s) School of Veterinary Medicine, University of Edinburgh, Scotland, UK; 2 YouthRISE Nigeria, Abuja, Nigeria; 3 Department of Medical Laboratory Science, Neuropsychiatric Hospital, Aro, Abeokuta, Nigeria; 4 Faculty of Medicine, Department of Public Health and Maritime Transport, University of Thessaly, Volos, Greece; 5 African Field Epidemiology Network, Abuja, Nigeria; 6 Genomic and Research Unit, Nigeria Centre for Disease Control and Prevention, Abuja, Nigeria; 7 Faculty of Medical Laboratory Science, Rivers State University, Port Harcourt, Nigeria; 8 Pathfinder International, Washington, USA; 9 Ahmadu Bello University, Zaria, Kaduna State Nigeria; 10 Nigerian Institute of Medical Research, Lagos, Nigeria

**Keywords:** HIV prevention, Pre-exposure prophylaxis (PrEP), Priority populations, Barriers, Facilitators, Health equity, PrEP uptake, Key populations

## Abstract

Purpose of the Review

This review examines the multifaceted barriers and facilitators influencing PrEP uptake among priority populations, including MSM, PWUD, sex workers, transgender individuals, and AGYW, with a focus on generating actionable recommendations for governments, providers, and community organisations to scale effective HIV prevention models globally.

Recent Findings

Despite PrEP's proven efficacy, uptake remains constrained by intersecting individual, provider, structural, and systemic barriers. Key challenges include limited awareness, misinformation, provider-level stigma, structural discrimination, legal repression, prohibitive costs, and healthcare inaccessibility, burdens that fall disproportionately on priority populations in low- and middle-income countries. Conversely, evidence from sub-Saharan Africa, Asia, Europe, and the Americas demonstrates that community-led awareness campaigns, integration of PrEP into sexual and reproductive health services, long-acting injectable formulations, mobile clinics, peer support structures, and digital health and telemedicine tools meaningfully improve access and adherence, particularly among youth and rural populations. Policy-level interventions, including decriminalisation of sex work, same-sex relationships, and drug use, alongside subsidised access and inclusion in universal health coverage frameworks, are identified as critical enablers of equitable reach.

Summary

Achieving global HIV prevention targets requires more than biomedical innovation. This review underscores that rights-based, community-embedded, and culturally affirming strategies paired with structural reform and sustained investment are essential to expanding PrEP access and equity. PrEP must be positioned not merely as a clinical tool, but as a cornerstone of an equity-driven HIV prevention architecture.

**Supplementary Information:**

The online version contains supplementary material available at 10.1007/s11904-026-00783-2.

## Introduction

Oral Pre-exposure prophylaxis (PrEP) is an effective biomedical intervention that utilizes antiretroviral therapies (ARTs) to lower the risk of acquiring HIV upon exposure among HIV-negative individuals [1]. It stands as one of the significant achievements resulting from decades of research aimed at preventing HIV infection, particularly in high-risk populations where behavior-based strategies have been less effective. Since the 2010 iPrEx trial demonstrated PrEP’s efficacy in preventing HIV infection, explicitly studying emtricitabine–tenofovir disoproxil fumarate (F/TDF) among men who have sex with men (MSM) [2] and its official approval for use by the US Food and Drug Administration in July 2012 [3] substantial progress has been made in the development and global use of PrEP. According to the Global PrEP Tracker, the total number of global PrEP initiations increased from approximately 100,000 in 2016 to over 8 million by 2024 [4, 5]. Specifically, in 2023, more than 3.5 million people received PrEP at least once, with over 75% of these individuals in the African region [6]. This figure reflects a 35% increase in the total number of people who received PrEP at least once worldwide between 2022 and 2023, clearly demonstrating increasing PrEP’s uptake, scale-up and coverage for HIV prevention worldwide [6]. Despite these notable and growing achievements, we are far from meeting UNAID’s PrEP goal of 21.2 million people using (initiating or continuing) PrEP globally in 2025 [7].

Evidence from large-scale systematic reviews and meta-analyses confirms that oral PrEP is both efficacious and effective across multiple populations at substantial risk of HIV infection. A meta-analysis of 15 randomized controlled trials involving over 25,000 participants found that oral tenofovir-based PrEP reduced HIV acquisition by 75% among men who have sex with men (MSM) and serodiscordant couples (RR = 0.25; 95% CI: 0.10–0.61), and by roughly 50% among people who inject drugs (PWID) (RR = 0.51; 95% CI: 0.29–0.92) [8]. Another systematic review covering 18 studies reported that trials with adherence above 70% achieved a 70% reduction in HIV incidence (RR = 0.30; 95% CI: 0.21–0.45), demonstrating a strong correlation between adherence and protection [8]. Both reviews found no evidence of behavioral risk compensation, minimal safety concerns, and rare occurrence of drug resistance among participants who initiated PrEP while acutely infected [8]. This evidence supports CDC’s claim that oral PrEP reduces HIV acquisition from sex by 99% and from injection drug use by ≥ 74% when taken as recommended [9].

While PrEP efficacy is well-documented, less attention has been given to the structural, behavioral, and policy-related barriers affecting uptake among priority populations globally, evidenced by the proportion of new HIV infections among priority populations [10]. We use the term *priority populations* to refer to the groups examined in this review (MSM, sex workers, transgender individuals, people who use drugs, and adolescent girls and young women) because each group exhibits elevated HIV vulnerability in many settings and because programmatic evidence supports their prioritization for PrEP scale-up. According to UNAIDS, priority populations account for 70% of new HIV infections globally [11]. Disaggregated data from the report showed that the incidence of new infections among people who use drugs (PWUD) (12%) and transgender individuals (TG) (6%) is higher than that observed in the general population [12]. These disparities underscore the urgent need to address systemic barriers to PrEP access among priority populations who are disproportionately affected. Several innovative programs and research efforts have successfully tackled these challenges by leveraging key facilitators of PrEP uptake.

Community-based delivery models, peer-led education, decentralized healthcare services, telemedicine, and differentiated service delivery approaches have all been shown to improve PrEP access and adherence [13, 14]. Integrating PrEP into primary healthcare settings, expanding same-day PrEP initiation, and reducing stigma through targeted awareness campaigns have also emerged as critical strategies for fostering PrEP adoption [14, 15]. This article aims to identify the predominant barriers limiting oral PrEP uptake among priority populations, explore the facilitators that enhance its accessibility and acceptance, and provide actionable recommendations for program implementation and policy development. We use the term *priority populations* to refer to the groups examined in this review (MSM, sex workers, transgender individuals, people who use drugs, and adolescent girls and young women) because each group exhibits elevated HIV vulnerability in many settings and because programmatic evidence supports their prioritization for PrEP scale-up.

## Methodology

We conducted a comprehensive literature review to identify barriers and facilitators of oral PrEP uptake among selected priority populations globally. Electronic searches were performed across PubMed, Scopus, and Google Scholar due to their broad coverage of peer-reviewed biomedical and public health literature. These databases were chosen for their relevance in indexing HIV prevention and implementation research. Searches utilized Medical Subject Headings (MeSH) terms for PubMed and relevant keywords for other databases. Core search terms included: “HIV,” “Pre-exposure prophylaxis,” “PrEP uptake,” “barriers,” “facilitators,” “key populations,” “priority populations,” “men who have sex with men,” “sex workers,” “transgender individuals,” “people who use drugs,” “adolescent girls and young women”. Boolean operators (AND/OR) were applied to refine results. Studies were eligible if they reported evidence on barriers or facilitators to oral PrEP uptake among any of the priority populations of interest.

We included peer-reviewed articles (original research, systematic reviews, meta-analyses), program evaluations, and relevant grey literature published in English, with no restriction on publication year, though preference was given to recent studies (2015–2024) to ensure currency of data. We excluded studies focusing exclusively on PrEP adherence, non-oral PrEP modalities, or populations outside the defined scope unless findings were broadly relevant to uptake. This review was conducted as a narrative synthesis rather than a systematic review, aiming to capture diverse evidence and emerging insights across global contexts. The lead author coordinated the screening of titles, abstracts, and full texts, with all authors contributing to different sections of the review. Where uncertainties or discrepancies arose, these were discussed among the author team and resolved by consensus.

Given the narrative nature of this review, a formal risk of bias assessment was not performed. This approach was deemed appropriate for capturing a wide range of evidence types, including both peer-reviewed and grey literature. We employed a narrative synthesis using a thematic analysis approach to group key ideas and findings into relevant themes. The extracted data were further succinctly discussed under appropriate headings to provide a clear and comprehensive overview of the study findings.

## Priority Populations in the Global HIV Epidemic: Risk Profiles and the Potential of Oral PrEP Uptake

The global HIV epidemic continues to disproportionately affect specific populations. In this study, we use the term *priority populations* to denote groups at elevated risk of HIV considered in this review (men who have sex with men; people who use drugs; sex workers (SW); transgender individuals; and adolescent girls and young women). This differs from the World Health Organization’s technical definition of *key populations* (men who have sex with men, people who inject drugs, people in prisons and other closed settings, sex workers and transgender people) and reflects our programmatic interest in including groups such as people who use drugs and adolescent girls and young women who face heightened vulnerability in many settings [16, 17]. These populations experience elevated vulnerabilities due to behavioral, biological, structural, and social factors that increase their risk of acquiring blood-borne infections, including HIV. Understanding the unique risk profiles of each population and the role of PrEP in reducing new infections is crucial to achieving global targets for HIV epidemic control Table 1.

**Table 1:** Definition of terms: priority populations vs. WHO key populations and rationale for inclusion in this study

Term	Definition	Examples	Rationale for Inclusion in This Review
WHO Key Populations (KPs)	Groups who, due to specific higher-risk behaviors and structural vulnerabilities, face disproportionately higher risk of HIV acquisition, and consistently lower access to services. WHO maintains a globally standard definition for programmatic focus.	Men who have sex with men (MSM) Sex workers People who inject drugs (PWID) Transgender people People in prisons and other closed settings	Used globally for standardization of HIV prevention programmes. WHO KPs are central to HIV policy, guidelines, funding streams, and monitoring frameworks. However, WHO KPs do not fully include all high-risk groups, especially those facing contextual or regional vulnerabilities not globally recognized.
Priority Populations (PPs)	A broader, context-dependent category that includes WHO Key Populations and additional groups who experience elevated HIV risk due to epidemiological, social, economic, or structural factors. Priority populations vary by country, epidemic type, and programmatic goals.	Adolescent girls and young women (AGYW) Fisherfolk Migrant or mobile workers Clients of sex workers Young people (15–24 years) in high-burden settings People in serodifferent couples People with high STI burden WHO Key Populations (MSM, SW, PWUD, TG people, prisoners)	Using the term “priority populations” reflects a more inclusive and epidemiologically accurate framing. It acknowledges: **1. Local epidemiology**: Certain groups (e.g., AGYW in sub-Saharan Africa, fisherfolk in East Africa) have HIV incidence *equal to or higher than WHO KPs* yet are not formally classified as KPs [18]. **2. Programmatic relevance**: National HIV programmes (e.g., PEPFAR, Global Fund, Nigeria’s NASCP) routinely designate additional groups as “priority populations” due to their contribution to incidence [19]. **3. Expanded scope of PrEP implementation**: PrEP is recommended for *all persons at substantial risk*, not only WHO KPs [20] **4. Avoiding exclusion**: Limiting analysis to WHO KPs would overlook significant high-risk groups central to global PrEP policy and practice. A paper reviewing national guidelines across 19 African countries found that even though WHO does not explicitly list AGYW as a key population for PrEP, **all 19 countries’ Country Operational Plans (COPs)** prioritized AGYW for PrEP access [19].

Furthermore, the review focused on priority populations with sufficient available literature on PrEP uptake barriers. Although priority populations are broad and context-specific, only groups with robust, comparable global evidence were included to maintain analytic coherence and methodological rigor.

### Men Who Have Sex with Men (MSM)

Men who have sex with men (MSM) continue to carry a disproportionately high burden of HIV globally, with UNAIDS estimating their risk of acquiring HIV to be 28 times higher than that of adult men in the general population [21], and in some countries, HIV prevalence in MSM exceeds 20–30%, even where general population prevalence is low [22]. This elevated epidemiological risk reflects the complex interaction of behavioural, biological, and structural determinants that shape vulnerability among MSM. Biologically, receptive anal intercourse carries a per-act transmission probability approximately 18 times higher than vaginal intercourse [23]. Behaviourally, inconsistent condom use, multiple partnerships, and high rates of sexually transmitted infections including rectal chlamydia and gonorrhoea, both of which significantly increase HIV acquisition risk contribute to sustained transmission [24]. These biological and behavioural factors are further compounded by structural conditions such as pervasive stigma, discrimination, and the criminalization of same-sex relationships across many countries, which continue to limit MSM’s access to appropriate and affirming healthcare services [25].

Evidence for PrEP efficacy among MSM is robust. Landmark trials such as PROUD in the United Kingdom and IPERGAY in France/Canada demonstrated over 85% reduction in HIV incidence among MSM using both daily and event-driven dosing [26–28]. Subsequent population-level data from Australia, London, and San Francisco showed rapid declines in new HIV diagnoses once PrEP was scaled up among MSM, highlighting strong real-world effectiveness [29–31]. These outcomes illustrate the good population-level effectiveness of PrEP and underscore its value not only as a biomedical intervention but also as an entry point to broader sexual health services, including STI screening, psychosocial support, and mental health care [32]. However, it is also important to state that even within high-income countries, disparities in oral PrEP uptake remain. Evidence from the United States shows that uptake has not been equitable across race, ethnicity, sex, and region, with Black and Latino MSM experiencing significantly lower access compared to White MSM despite comparable levels of HIV risk [33].

In in low- and middle-income countries (LMICs), oral PrEP uptake remains substantially lower [34]. Specifically, stigma, discrimination, provider bias, police harassment, and fear of being “outed” continue to inhibit service uptake [35–37]. Emerging evidence also suggests that MSM in LMICs experience lower levels of PrEP persistence, with discontinuation often driven by mobility, inconsistent risk perception, and structural insecurity [34]. Significant research gaps persist, particularly regarding how best to deliver PrEP in criminalized environments, how to sustain long-term adherence among MSM outside high-income regions, and how differentiated or community-led models might enhance service acceptability in LMIC contexts [38–40]. Addressing these gaps is essential for ensuring that PrEP achieves its full potential among MSM globally and for closing persistent inequities in HIV prevention.

### People Who Use Drugs (PWUD)

People who use drugs (PWUD) remain a population at markedly elevated risk of HIV acquisition, with people who inject drugs (PWID) facing an estimated 22-fold greater HIV risk than the general population, driven principally by sharing of injecting equipment and limited access to sterile supplies [41]. Non-injecting drug users also experience elevated risk through overlapping sexual vulnerabilities, including transactional sex and concurrent partnerships [42]. The regional contribution of PWUD/PWID to new infections is heterogeneous: PWID drive large epidemics in parts of Eastern Europe and Central Asia; the role of PWID in West Africa is more varied and, in many settings, is less dominant [43, 44].

Despite this high and preventable risk, PWUD and PWID in particular have been underserved in PrEP programming [45]. Historically, much PrEP implementation has prioritized sexual transmission pathways; consequently, fewer programmes have integrated PrEP into harm-reduction platforms that serve PWID. The Lancet Bangkok Tenofovir Study a randomized, placebo-controlled trial among PWID provided the first rigorous evidence that daily oral tenofovir reduced HIV incidence in an injecting population when delivered alongside comprehensive harm-reduction services (including needle-syringe programmes and opioid substitution therapy) [46]. This trial, together with pooled analyses and later implementation studies, supports the efficacy and potential effectiveness of PrEP for PWID when combined with established harm-reduction interventions [47, 48].

Operational evidence suggests that PrEP is most effective for PWID when integrated within comprehensive, low-threshold harm-reduction packages. Programmes that colocate PrEP with needle-syringe distribution, opioid substitution therapy (OST), hepatitis C diagnosis and treatment, and community-led outreach have achieved better uptake and retention than facility-based, stand-alone models [49]. Key evidence gaps remain and few implementation studies quantify the marginal benefit of adding PrEP to mature harm-reduction programmes under real-world constraints [10, 50] More implementation science is needed to measure cost-effectiveness and budget impact of scaling PrEP for PWID in low-resource settings where donor support is variable.

### Sex Workers

Sex workers (SW), particularly female sex workers (FSW), experience a substantially elevated HIV burden, with evidence indicating that FSW face approximately a 13-fold higher risk of acquiring HIV compared to adult women in the general population [51]. Although female sex workers (FSW) remain the most studied group, male and transgender sex workers face comparable and in some contexts even more severe structural disadvantage, including pervasive stigma, punitive legal environments, police harassment, violence, and widespread exclusion from healthcare systems [52]. These intersecting vulnerabilities shape HIV risk across all sex worker groups, though the ways they manifest may differ by gender identity, work setting, and social visibility.

Risk among sex workers is driven not only by sexual exposure to multiple partners and inconsistent condom use but also by structural determinants that limit their negotiating power for safer sex [51]. High rates of physical and sexual violence often from clients, intimate partners, or law enforcement further constrain agency and elevate HIV vulnerability [53]. Mobility, which is an intrinsic feature of sex work in many regions, plays a critical and often under-recognised role in shaping risk [54]. Sex workers frequently move across urban hotspots, border towns, mining sites, transport corridors, and seasonal economic hubs [55, 56]. This mobility disrupts continuity in healthcare access, weakens social support networks, and complicates sustained engagement with HIV prevention programmes [55, 56]. Mobility undermines continuity of care by interrupting follow-up visits and medication refills and by exposing sex workers to novel client pools and variable policing that reduce service engagement [55, 56].

Evidence on the efficacy and effectiveness of pre-exposure prophylaxis (PrEP) among sex workers continues to grow. Clinical and implementation data demonstrate that PrEP is highly efficacious when taken consistently, and programmes have shown positive results in diverse sex worker populations [57, 58]. The South African TAPS Demonstration Project revealed high interest in PrEP among FSW [58] and adherence levels sufficient to confer protection, while programmes in Kenya and Zimbabwe have similarly reported encouraging uptake and retention [58]. However, real-world effectiveness has not been substantiated in literature. Also, uptake remains low in many regions due to stigma, legal barriers, and the transient nature of sex work [59]. Successful implementation hinges on tailoring PrEP services to be mobile, confidential, and integrated into broader sexual and reproductive health services [60]. Peer-led outreach and community empowerment strategies have also been vital in increasing PrEP literacy and uptake [61]. Significant research gaps remain. Evidence on PrEP implementation among male and transgender sex workers is comparatively sparse, despite these groups often reporting higher levels of violence, policing, and economic insecurity.

### Transgender Individuals

Transgender people, particularly transgender women, are among the most marginalized and vulnerable to HIV [62]. Transgender women are estimated to be 49 times more likely to acquire HIV compared to the general population [63]. This astronomical risk is driven by multiple factors: high prevalence of sex work, social exclusion, gender-based violence, lack of legal recognition, and barriers to accessing gender-affirming and HIV services [64]. Globally, transgender individuals have been inadequately represented in clinical trials and often subsumed within MSM categories, which fail to capture their unique needs [65]. However, studies such as iFACT in Thailand and the TRIUMPH project in the U.S. have provided evidence that transgender women can successfully use PrEP when offered in gender-affirming environments [66, 67]. Tailoring PrEP services to be affirming of gender identity and inclusive in delivery is critical to addressing the epidemic among this group. Important research gaps persist, including limited disaggregated data on transgender men, evolving but still incomplete evidence on the interactions between feminizing hormone regimens and PrEP pharmacokinetics, insufficient exploration of PrEP acceptability among transgender communities, and a lack of large-scale PrEP demonstration projects in low- and middle-income countries where transgender HIV burden is increasing [65].

### Adolescent Girls and Young Women (AGYW)

Adolescent girls and young women (AGYW) in sub-Saharan Africa bear a disproportionate burden of HIV, accounting for a significant share of new infections [16]. In some countries, they represent more than 60% of all new cases despite constituting a much smaller proportion of the population [16]. This elevated risk is shaped by intersecting vulnerabilities, including entrenched gender inequality, intergenerational sexual relationships, limited access to education and sexual and reproductive health services, restrictive social norms that reduce autonomy and agency in sexual decision-making, as well as biological factors such as higher prevalence of genital inflammation and sexually transmitted infections, which increase susceptibility to HIV [68–70].

Evidence has demonstrated that oral PrEP is efficacious for AGYW when adherence is optimal, providing substantial protection against HIV acquisition. Programmatic data from large initiatives such as DREAMS (Determined, Resilient, Empowered, AIDS-free, Mentored and Safe) show that integrating oral PrEP within a layered prevention package alongside empowerment, violence reduction, and economic interventions supports its effective use [71]. Implementation studies further highlight that when AGYW receive youth-friendly, confidential, and community-supported services, they can successfully initiate and adhere to PrEP [71]. Despite these successes, uptake in routine settings remains constrained by low risk perception, stigma, misconceptions about side effects, and broader structural barriers.

Furthermore, program coverage remains uneven, and many countries have yet to fully scale youth-centered PrEP delivery models [72]. Research gaps persist around optimizing PrEP delivery for adolescent subgroups, understanding the role of parental consent laws, and evaluating how long-acting modalities will influence adherence, continuation, and real-world effectiveness in this population.

In closing this section, it is important to state that oral PrEP has emerged as a transformative tool in the global HIV prevention landscape. However, its full potential will only be realized when its delivery is tailored to the unique needs of priority populations. Addressing the biological, behavioral, and structural factors that drive risk among MSM, PWUD, sex workers, transgender individuals, and AGYW is essential. Equally critical is ensuring that PrEP is embedded within comprehensive, rights-based health systems that affirm the dignity and agency of these communities. As global health efforts move toward ending AIDS as a public health threat, centering priority populations in PrEP programming is not just strategic but imperative.

## Barriers Influencing Oral PrEP Uptake Among Priority Populations at Risk of HIV Across the Globe: A Socio-Ecological Model

Oral PrEP uptake is constrained by a constellation of interlinked barriers operating across individual, interpersonal, community, institutional, and structural levels, each shaping how priority populations perceive, access, and initiate PrEP within diverse global contexts. Using a socio-ecological model adapted from Bronfenbrenner [73] and McLeroy [74] we discuss these barriers in six distinct levels.

### Individual-Level Barriers

At the individual level, limited awareness and misinformation remain primary obstacles to PrEP uptake across global priority populations. Awareness of PrEP is still unevenly distributed; in China, knowledge remains low among men who have sex with men (MSM) irrespective of their sexual orientation [75], while in Europe, female priority populations exhibit similarly low awareness due to systemic neglect and stigma in prevention programming [76]. Among adolescents and young adults, awareness remains particularly poor, with studies from North America showing low levels of knowledge among high-risk youth [77]. In Tanzania, only 7% of young adults reported any awareness of PrEP, and willingness to use it was equally low [78]. Individual factors such as educational attainment also play an important role; studies among Black MSM in the United States show that those with higher education levels are more likely to be aware of PrEP and its benefits [79, 80].

Misconceptions further complicate uptake, including the persistent belief that PrEP is equivalent to antiretroviral therapy (ART), which can generate fears of HIV-related stigma if others assume its users are HIV-positive [81]. Among transgender women, concerns about potential interactions between PrEP and hormone therapy discourage initiation and contribute to mistrust of biomedical HIV prevention services [82]. These individual factors interact to produce conditions in which many people who might benefit from PrEP do not consider themselves appropriate candidates for it or do not feel equipped to initiate it.

### Interpersonal-Level Barriers

Interpersonal dynamics including partner relationships, family influences, peer networks and direct patient–provider interactions strongly affect PrEP initiation. Experiences of stigma within families or peer groups can discourage individuals from adopting PrEP, particularly in settings where taking PrEP is equated with risky sexual behavior or assumed HIV infection [81]. Provider–client interactions fueled by training gaps and negative attitudes represent another critical interpersonal barrier [83]. Evidence from multiple settings demonstrates that ethnic and racial biases among healthcare providers shape communication quality, treatment outcomes, and clients’ trust in health services [84, 85]. Among people who use drugs, healthcare workers’ moralizing attitudes, including reluctance to provide PrEP or refer individuals for PrEP services, can significantly reduce uptake [86]. Interpersonal environments therefore create reinforcing or discouraging conditions that profoundly shape clients’ motivation and willingness to initiate PrEP. These include homophobia, transphobia, and discrimination directed at sex workers and people who use drugs, which further erode trust in providers and discourage individuals from seeking PrEP services [87].

### Community-Level Barriers

At the community level, prevailing norms, social stigma and mobility patterns significantly influence PrEP initiation. In many contexts, PrEP use is associated with stereotypes and moral judgments that frame it as a marker of promiscuity or deviant sexual behavior, discouraging individuals from seeking it [38, 88, 89]. Such community-level stigma is particularly pronounced in conservative settings, where fear of being seen at PrEP clinics can prevent individuals from accessing services. In China, for instance, the positioning of PrEP delivery within HIV clinics creates a major deterrent, as being observed there may inadvertently disclose one’s sexual orientation or perceived HIV status [68, 90].

Highly mobile populations such as sex workers face community-level barriers related to their livelihood patterns. Frequent movement across hotspots and border regions often limits their ability to access local PrEP services and undermines opportunities for counseling or initiation [91]. Additionally, the absence of community-based HIV prevention infrastructure in some settings restricts opportunities for outreach, meaning many individuals never encounter accurate information or accessible pathways to begin PrEP [92]. Furthermore, community barriers also include limited autonomy in health decision-making [93]. In many contexts, AGYW must seek permission from parents, partners, or guardians to access services, and age restrictions on sexual and reproductive health services further constrain their ability to initiate PrEP [94]. These community factors collectively shape the broader social environment in ways that may either support or obstruct uptake.

### Health System and Institutional Barriers

Institutional barriers within healthcare systems remain some of the most persistent obstacles to PrEP initiation. In several countries, PrEP services are centralized in urban referral centers or specialized HIV clinics, limiting access for rural populations and reinforcing fears of stigma. In France, such centralization has disproportionately affected people outside major urban areas [95], while in other settings, including China, limiting PrEP delivery to HIV specialty clinics has created strong deterrents to initial uptake [90].

Health workforce limitations including insufficient training, negative provider attitudes, and uncertainty about who is responsible for prescribing PrEP further restrict access to initiation [96]. Specifically, limited provider knowledge also indirectly influences individual decisions. For instance, inadequate understanding of PrEP among healthcare workers in Iran contributes to insufficient counseling and reduces client confidence in initiating PrEP [14]. This “purview paradox,” in which HIV specialists rarely see HIV-negative individuals who need PrEP and primary-care providers often feel unprepared to prescribe it, creates significant gaps in service delivery [97, 98]. Insufficient staffing, chronic shortages of trained providers, and inconsistent provider knowledge also reduce opportunities for potential users to receive accurate counseling [14].

Operational constraints also limit initiation. Long waiting times, restricted clinic hours, transportation challenges, and lack of confidentiality within service settings discourage clients from engaging with prevention services [99, 100]. Stock-outs and intermittent supply of PrEP commodities documented in several LMIC health facilities [97, 101] further reduce client motivation to begin PrEP, as inconsistent availability undermines trust in the health system’s ability to support prevention needs. While integration of PrEP into HIV specialty clinics has created barriers by reinforcing stigma and limiting accessibility, integration into broader health services (e.g., reproductive health, primary care, harm reduction programs) has been shown to facilitate uptake. This duality underscores the importance of context and service design in determining whether integration acts as a barrier or an enabler.

These institutional challenges collectively restrict access to convenient, acceptable, and client-friendly pathways to PrEP initiation.

### Structural and Policy-Level Barriers

Structural and policy-level factors create powerful upstream barriers to PrEP uptake. Criminalization of priority populations including same-sex relations, sex work, and drug use remains one of the most critical obstacles across Africa and Asia. In Zambia, fear of criminalization has discouraged priority populations from accessing health facilities, even when dealing with healthcare workers [102]. In Nigeria, the Same-Sex Marriage (Prohibition) Act has further entrenched stigma and driven MSM and transgender people away from HIV prevention services [103]. Across Southeast Asia, punitive environments similarly limit the willingness of priority populations to seek PrEP services [104, 105].

Legal restrictions affecting civil society also pose significant challenges. Prosecution of community-based organizations reduces outreach efforts, hinders distribution of accurate health information, and weakens national HIV prevention infrastructure [102]. Financial barriers, including the high cost of medication, laboratory tests, and clinical visits, continue to restrict uptake among uninsured or low-income individuals in both high-income and low-income countries [106–108]. Persistent inequities in health-financing systems also contribute to the emergence of “PrEP deserts,” geographical areas where PrEP services are either nonexistent or inaccessible [109]. Broad structural inequalities, including institutional racism, gender inequalities, and poverty, further pattern disparities in access among marginalized groups in LMICs Table 2.

**Table 2:** Highlights of barriers to oral PrEP uptake among priority populations using the socio-ecological model

Socio-Ecological Level	Key Barriers	Summary of Key Issues Identified Across Studies	Key Citations
Individual-Level Barriers	• Low awareness and knowledge of PrEP • Misconceptions and fear of side effects • Low self-perceived HIV risk • Competing priorities • Internalized stigma	Awareness remains low among MSM in China and among women in Europe, with extremely limited knowledge among young adults in Tanzania and high-risk youth in North America. Misconceptions such as equating PrEP with ART drive fear of stigma. Transgender women express concerns about interactions with hormone therapy.	[14, 75–82]
Interpersonal-Level Barriers	• Partner disapproval or mistrust • Fear of IPV or conflict • Peer discouragement • Family stigma • Poor provider–client interactions	Family and peer stigma discourage uptake where PrEP is framed as evidence of risky behavior or assumed HIV infection. Provider–client bias and poor communication especially racial/ethnic bias reduce trust and willingness to start PrEP. Among people who use drugs, moralizing attitudes from providers reduce referrals and uptake.	[81, 84–86]
Community-Level Barriers	• HIV and PrEP stigma in communities • Cultural/religious norms • Limited community-based outreach • Mobility of priority populations • Fear of being seen at HIV clinics	Community stigma frames PrEP as promiscuity or HIV infection, deterring access. In China, locating PrEP in HIV clinics deters MSM due to fear of outing. Mobile populations such as sex workers struggle with access due to movement across hotspots. Weak community HIV prevention infrastructure limits outreach.	[38, 68, 89–92, 110]
Health System & Institutional Barriers	• Long waiting times and inconvenient hours • Stockouts and supply issues • Fragmented or centralized PrEP service delivery • Lack of KP- and youth-friendly services	Centralization of PrEP in referral or HIV clinics (e.g., France, China) restricts access and reinforces stigma. Providers often lack PrEP training or hold negative attitudes. The “purview paradox” limits prescribing. Long travel times, clinic delays, and weak confidentiality discourage access. Stockouts in LMIC facilities undermine trust in prevention services. Limited provider knowledge (e.g., Iran) reduces client confidence.	[14, 16, 90, 95, 97–100]
Structural & Policy-Level Barriers	• Criminalization of same-sex relations, sex work, drug use • Restrictive policies • Poverty and economic hardship • Gender inequality • Limited national rollout or funding gaps	Criminalization such as in Zambia and Nigeria drives MSM, sex workers, and PWUD away from services. Southeast Asia’s punitive legal climate limits willingness to seek PrEP. Financial barriers persist across income settings. “PrEP deserts” emerge in underfunded regions. Structural racism, gender inequity, and poverty further restrict access.	[102–109, 111, 112]
Intersectionality of Barriers	• Combined legal, social, economic, and health-system barriers • Compounded disadvantage for marginalized populations	Barriers at different levels reinforce each other. Criminalization, community stigma, and discriminatory providers create layered deterrents for MSM, sex workers, and PWUD. Poverty and structural inequalities intersect with weak health systems, reducing uptake among those facing multiple forms of vulnerability.	

### Intersectionality of Barriers

Barriers across these socio-ecological levels rarely occur in isolation. Instead, they interact to create compounded disadvantages that significantly suppress PrEP uptake among individuals who experience multiple vulnerabilities simultaneously. For example, criminalization at the policy level, combined with community stigma and provider discrimination, produces layered deterrents for MSM, sex workers, and people who use drugs, reducing both motivation and opportunity to initiate PrEP [14]. Similarly, poverty interacts with the structural centralization of services and health-system constraints to further restrict the ability of marginalized individuals to access healthcare services that may include PrEP initiation [113]. Evidence increasingly shows that these barriers reinforce each other, producing environments in which certain groups particularly those facing overlapping social, legal, and economic exclusion are systematically less likely to initiate PrEP [114].

## Facilitators of Oral PrEP Uptake Among Priority Populations Worldwide

Facilitators of oral PrEP uptake among priority populations worldwide span a diverse range of programmatic, community-led, and structural interventions that strengthen awareness, accessibility, trust, and policy environments, collectively enabling higher initiation and sustained use of PrEP across different contexts. We have discussed these enablers of oral PrEP uptake among the listed priority population in this study under sex themes.

### Education and Awareness Campaigns

PrEP is a vital HIV prevention strategy, yet uptake among priority populations varies globally. Education and awareness campaigns are key facilitators, addressing knowledge gaps and stigma across diverse regions. For example, in South Africa, the Desmond Tutu Health Foundation implemented a community-led campaign disseminating PrEP information among MSM and transgender women in Cape Town, increasing uptake by 40% within six months [115]. Brazil’s “PrEP Brasil” campaign combined public health messaging with Carnival festivities, contributing to a 25% rise in PrEP prescriptions among MSM in Rio de Janeiro [116]. In Peru, focus groups with MSM and transgender persons revealed that education on PrEP’s efficacy and safety significantly raised acceptability [117]. Also, qualitative research from Zimbabwe highlights that privacy, confidential/free services, supportive health-worker attitudes, and education of parents/partners are central to improving PrEP uptake among which reinforce the need for youth-friendly, stigma-aware education strategies [118].

Community-driven campaigns in trusted venues, such as syringe service programs, and digital tools like SMS reminders have shown promise in high-income settings [119], though evidence from low- and middle-income countries remains limited. In high-income settings, syringe service programs (SSPs) have been effective venues for PrEP engagement although these studies refer long-acting injectable PrEP, SSP-based surveys show low baseline awareness and substantial interest in linkage indicating that low-threshold, harm-reduction-aligned education can be promising for PWID [120]. In Nigeria, a mixed-methods study revealed that MSM preferred receiving PrEP information through social media channels such as WhatsApp, Facebook, and Instagram, emphasizing the need for digital outreach tailored to specific KP preferences [121]. A systematic review study in Nigeria also demonstrate substantial demand: among FSWs, willingness to use PrEP was high in Lagos (80%), Anambra (91%), and Kano (66.8%), though awareness and practice vary, highlighting how culturally sensitive education can increase PrEP initiation among sex workers [122].

Across these settings, culturally sensitive campaigns delivered by community-based organizations consistently enhance acceptance and uptake. Challenges remain, including reaching marginalized subgroups and countering misinformation. However, the evidence indicates that education is a foundational facilitator.

### Integration with Other Services

Integrating HIV PrEP into reproductive health services has become a critical strategy for expanding access to HIV prevention, particularly for high-risk populations. While integration of PrEP into HIV specialty clinics has created barriers by reinforcing stigma and limiting accessibility, integration into broader health services (e.g., reproductive health, primary care, harm reduction programs) has been shown to facilitate uptake [94]. This duality underscores the importance of context and service design in determining whether integration acts as a barrier or an enabler.

STI clinics and reproductive health services are well-positioned to provide PrEP, as they regularly engage with individuals at an increased risk of HIV [123]. This approach not only enhances accessibility but also makes HIV prevention more acceptable by addressing multiple health needs within a single setting [124]. There is significant evidence of increased PrEP use among clients when incorporated into broader sexual and reproductive health programs [125–128].Integration of PrEP with SRH has been proven to improve the effective use of PrEP [129], which can also generate other benefits such as opening up opportunities to promote both HIV and SRH services [130].

In Kenya, a pragmatic stepped-wedge randomised trial evaluated the integration of PrEP services into public HIV care clinics serving at-risk groups. The study demonstrated sustained high monthly uptake of PrEP, indicating that public HIV care clinics are a feasible setting for delivering integrated PrEP services. The clinics effectively utilized their existing infrastructure and workforce to implement PrEP services with high fidelity. Notably, individuals with higher HIV risk scores at baseline were more likely to continue PrEP use beyond three months, suggesting alignment of PrEP use with periods of heightened HIV risk. The study concluded that integrating PrEP into public health facilities is feasible and can be achieved with high fidelity when healthcare providers receive appropriate training and technical support [131].

In addition, the PROUD study, conducted in the UK, was a pragmatic randomized trial within sexual health services. It evaluated the effectiveness of PrEP in preventing HIV-1 infection among MSM. This open-label trial enrolled 544 participants, assigning them to either immediate PrEP initiation or deferred PrEP after 12 months. The findings revealed a significant reduction in HIV incidence among the immediate PrEP group, with an 86% relative reduction compared to the deferred group. This study underscores the efficacy of PrEP in real-world settings and highlights the importance of integrating PrEP into sexual health services to curb HIV transmission among high-risk populations [28].

Evidence also supports integration of PrEP with gender-affirming care for transgender women and with SRH services for female sex workers in various contexts [132, 133]. Additionally, integration with harm reduction programs such as opioid substitution therapy and syringe service programs has shown promise in improving PrEP uptake among people who use drugs [10, 134]. Across these models, integration enhances accessibility, acceptability, and continuity of care, making it a viable approach for scaling up PrEP services. However, gaps remain in certain regions and service delivery models, underscoring the need for more targeted research to evaluate effectiveness among priority populations and ensure interventions are tailored to their unique needs [135, 136].

### Innovations in PrEP Delivery

Innovations in oral PrEP delivery have focused on improving accessibility, convenience, and adherence through differentiated service delivery (DSD) models and digital health solutions. Pharmacy-led PrEP programs have emerged as a promising strategy to expand access beyond traditional clinical settings. In the United States, states such as California and Colorado allow pharmacists to initiate and dispense PrEP without a prior physician visit, increasing convenience for individuals who face stigma or logistical challenges in accessing care [137].Similar models are being piloted in South Africa and Kenya to reach priority populations, including MSM and sex workers [138–140].

Community health workers (CHWs) also play a critical role in delivering PrEP in resource-limited settings. CHW-led models reduce reliance on facility-based care and bring PrEP closer to communities, particularly for female sex workers and people who use drugs [134, 141–143]. Demedicalization through DSD approaches such as multi-month dispensing, community-based refills, and peer-led distribution has been shown to improve retention and adherence among MSM and transgender women [144].

In addition to adherence-focused DSD models, uptake-focused strategies such as same-day PrEP initiation have proven effective in reducing attrition between eligibility screening and initiation [145]. Evidence from multiple settings shows that offering PrEP immediately after HIV testing or risk assessment significantly increases uptake, particularly among AGYW, MSM, and sex workers [146–148].These approaches highlight how DSD can be tailored not only to support continuation but also to facilitate rapid initiation.

Telemedicine platforms have expanded PrEP access by enabling remote consultations and follow-up. Programs like Iowa TelePrEP, PrEPTech, Nurx, and PlushCare utilize video, telephone, and text messaging for consultations, with laboratory testing conducted through local facilities or via at-home kits [149]. These programs report high retention rates (76–98.7% at 6 months) and PrEP initiation rates (84–94% after first appointments), indicating their potential to support adherence.

Mobile health (mHealth) apps also offer innovative adherence support. For example, “Project Raincoat” was piloted among young MSM and young transgender women in Thailand [150]. Based on the information–motivation–behavioral skills model, the app featured risk assessments, customizable reminders, and rewards. While 87% of participants used the app, adherence was higher among frequent users (59–67% at 3–6 months), though not statistically significant compared to controls [150]. Engagement declined over time, highlighting the need for sustained user interaction strategies. These innovations collectively demonstrate how oral PrEP delivery can be adapted to meet the needs of diverse populations and overcome barriers related to stigma, distance, and health system constraints.

### Peer and Community Support

Peer- and community-led support has emerged as a critical facilitator of PrEP uptake among priority populations (KPs), who often experience structural and social marginalization, leading to mistrust of formal health systems and limited access to HIV prevention tools [14, 38, 151]. Evidence from multiple regions underscores the impact of peer-led interventions on PrEP uptake [152–155].

In Thailand, the Princess PrEP programme, implemented by community-based organizations, demonstrated that trained lay providers could successfully initiate and monitor PrEP among men who have sex with men (MSM) and transgender women, resulting in a scale-up of community-based PrEP services across the country [156]. Similarly, in Kenya and South Africa, programs such as the DREAMS initiative and the Community PrEP Study have highlighted how peer educators within adolescent girls and young women (AGYW) communities have helped increase awareness, demand, and adherence to PrEP through girl-led clubs and community dialogues [157].

Importantly, community-led organizations also shape service delivery environments to be more accessible and affirming. Mobile clinics, drop-in centres, and pop-up PrEP campaigns, designed and run by KPs, are increasingly recognized as best practices [158]. In Ukraine, despite the ongoing conflict, community groups have integrated HIV prevention services into harm reduction programs for people who use drugs (PWUD), demonstrating resilience and innovation in reaching high-risk groups. These examples reinforce that meaningful engagement with KP-led groups can extend the reach of PrEP into hard-to-reach and criminalized populations. Peer and community support strengthens the PrEP continuum from awareness to adherence. Global experiences confirm that trust, cultural alignment, and peer empowerment are not optional add-ons but essential elements of successful PrEP programming. Scaling up community-led interventions with long-term, sustainable funding and technical support should be prioritized in global HIV prevention strategies, especially where traditional systems have historically failed marginalized populations [159].

### Policy and Funding Support

Structural enablers such as progressive policy reform and sustained financial investment are vital to increasing PrEP accessibility, affordability, and uptake among these groups. Globally, legal frameworks remain a significant barrier to PrEP access. In over 70 countries, same-sex relations are criminalized, discouraging MSM from accessing sexual health services, including PrEP. Similarly, the criminalization of sex work and drug use contributes to the surveillance and policing of communities rather than the delivery of care [160]. While the broader discussion of criminalization is addressed in the Barriers section, here we emphasize that policy shifts can have a dramatic impact on HIV prevention outcomes. For example, a study published in *The Lancet HIV* found that decriminalization of sex work could significantly reduce HIV incidence by creating safer, more enabling environments for prevention [161].

National policy integration has also proven effective. Brazil’s decision to integrate PrEP into its national health system and offer it free of charge to priority populations resulted in rapid uptake among MSM and transgender women, especially in urban centers [162]. Likewise, South Africa’s adoption of national PrEP guidelines along with a decentralized delivery model through primary care and community health workers demonstrated how supportive governance can make PrEP part of routine care for AGYW and sex workers [163–165].

Financing mechanisms remain critical. In high-income countries, out-of-pocket expenses and insurance barriers have hindered PrEP uptake, particularly among uninsured and underinsured racial and ethnic minorities [165–167]. In contrast, donor-funded programs in sub-Saharan Africa, such as PEPFAR and the Global Fund, have enabled PrEP delivery at scale through national rollouts, particularly among adolescents and female sex workers. However, sustainability remains a concern, especially in middle-income countries transitioning from donor support. Global advocacy efforts are increasingly calling for PrEP to be classified as an essential health commodity. Doing so would ensure inclusion in universal health coverage (UHC) packages and national formularies, making PrEP procurement and distribution more consistent [168].

Furthermore, community-led policy monitoring, where priority populations document service gaps and advocate for legal reform, has proven effective in countries such as Nepal, Ukraine, and Kenya, where sex workers and transgender individuals have successfully lobbied for policy inclusion in national PrEP strategies [169, 170]. To achieve the ambitious targets for HIV prevention outlined in UNAIDS’ 2025 roadmap, structural enablers must be addressed with the same urgency as individual behavior change. Legal reforms that dismantle punitive laws and protect the rights of priority populations, coupled with reliable financing mechanisms for PrEP access, will determine whether the global HIV response leaves no one behind.

## Conclusion and Recommendations

This global review underscores the transformative potential of PrEP in reducing HIV transmission among priority populations, including MSM, PWUD, sex workers, transgender individuals, and AGYW. While the efficacy of PrEP is well-established, its global uptake remains hindered by complex, interrelated barriers, including stigma, legal and policy constraints, socioeconomic challenges, cultural perceptions, and infrastructural limitations. These barriers disproportionately affect vulnerable and marginalized groups, especially in LMICs.

Several facilitators have proven effective in promoting PrEP uptake: community-led outreach, integration with other health services, innovations in oral PrEP delivery (e.g., pharmacy-based models, telehealth, DSD approaches), and enabling policy environments. In addition, emerging PrEP modalities such as long-acting injectables, implants, and vaginal rings represent promising future options for individuals who struggle with daily oral regimens [161]. Evidence from trials of long-acting cabotegravir demonstrates high efficacy and acceptability among MSM and women [171], while studies of the dapivirine vaginal ring show feasibility and potential to expand choice for AGYW in sub-Saharan Africa [172]. Notably, the ground-breaking lenacapavir innovation is expected to provide new evidence on the impact of alternatives to oral PrEP [173]. These modalities could improve adherence and expand choice, but they also raise concerns about re-medicalization, as they often require clinical administration.” Researchers and implementers should anticipate this shift by exploring strategies to maintain accessibility and equity as new technologies scale up.

Recent U.S. funding cuts threaten global HIV prevention programs and may reduce PrEP access for priority populations. Sustaining and accelerating PrEP gains will require diversified financing, domestic resource mobilization, and strong policy commitments to integrate PrEP into universal health coverage (UHC) packages.

These recommendations are directed at multiple stakeholder including policy-makers, implementers, providers, researchers, and community organizations who collectively shape the PrEP landscape. Action across these groups is essential to overcome barriers and accelerate uptake among priority populations.

- Policy: Enact and enforce anti-discrimination laws, decriminalize priority populations, and classify PrEP as an essential health commodity for inclusion in UHC packages.
- Delivery: Decentralize PrEP services through task-shifting, pharmacy-based models, and differentiated service delivery approaches, including multi-month dispensing and community-based refills.
- Integration: Embed PrEP within sexual and reproductive health services and harm reduction programs to reach underserved populations.
- Community Engagement: Strengthen peer-led models, mobile clinics, and digital health interventions to build trust and address stigma.
- Funding: Secure sustainable financing through domestic budgets and global partnerships to offset donor dependency and mitigate the impact of funding cuts.
- Research: While many studies on new PrEP modalities and integration strategies are ongoing, future research should focus on implementation science, cost‑effectiveness, and psychosocial factors influencing PrEP uptake and continuation, particularly in hard‑to‑reach populations.

Ending the HIV epidemic requires more than biomedical solutions; it demands equity, empowerment, and a reimagined approach to HIV prevention that centers on the lived realities of priority populations. A global commitment to addressing structural barriers and scaling up what works primarily through community engagement and inclusive policies will be vital to ensuring that PrEP reaches its full potential.

## Supplementary Information

Below is the link to the electronic supplementary material.

Supplementary File 1 (DOCX 218 KB)

## Data Availability

Not applicable because no new data or databases were used in the preparation of this work.
