# Supplementary material for: Barriers and Facilitators of Oral PrEP Uptake Against HIV Among Selected Priority Populations: A Global Perspective
Source: Curr HIV/AIDS Rep. 2026 Mar 30;23(1):14. doi: 10.1007/s11904-026-00783-2 (PMC13035875; doi:10.1007/s11904-026-00783-2)
Supplement: Supplementary file 1 — (DOCX 218 KB) [file 11904_2026_783_MOESM1_ESM.docx]

**Important information. Please read.**

- This form should be used by authors to request any change in authorship (adding/deleting authors) including changes in corresponding authors. This form should not be used for name changes. Please fully complete all sections. Use black ink and block capitals and provide each author’s full name with the given name first followed by the family name.
- By signing this declaration, all authors guarantee that the order of the authors are in accordance with their scientific contribution, if applicable as different conventions apply per discipline, and that only authors have been added who made a meaningful contribution to the work.
- Please note, in author collaborations where there is formal agreement for representing the collaboration, it is sufficient for the representative or legal guarantor (usually the corresponding author) to complete and sign the Authorship Change Form on behalf of all authors, **next to the added/removed author(s)**. **(Complete Section 3, followed by Section 6.)**

In author collaborations where there is no formal agreement for representing the collaboration and **there are more than 10 authors**, one may sign for all, provided the signer appends correspondence that attests that each of the authors have agreed to the change **and the added/removed authors sign the form**. **(Complete Section 3, followed by Section 6.)**

- Please note, we cannot investigate or mediate any authorship disputes. If you are unable to obtain agreement from all authors (including those who you wish to be removed) you must refer the matter to your institution(s) for investigation. Please inform us if you need to do this.
- If you are not able to return a fully completed form within **30 days** of the date that it was sent to the author requesting the change, we may have to withdraw your manuscript. We cannot publish manuscripts where authorship has not been agreed by all authors (including those who have been removed).
- Incomplete forms will be rejected.
- Please return/upload this form, fully completed, to the Journals Editorial Office. The Journal and/or Publisher will consider the information you have provided to decide whether to approve the proposed change in authorship. We may decide to contact your institution for more information or undertake a further investigation, if appropriate, before making a final decision.

| **Section 1: Please provide the current title of manuscript** | **Manuscript ID no.:** 1d625e08-6bb0-4f6a-b442-f5923a5f6049 v3.1 |
| --- | --- |
| Title: Barriers and Facilitators of Oral PrEP Uptake Against HIV Among Selected Priority Populations: A Global Perspective | |

**Section 2: Please provide the previous authorship, in the order shown on the manuscript before the changes were introduced. Please indicate the corresponding author by adding (CA) behind the name.**

|  | First name(s) | Family name | ORCID or SCOPUS id, if available |
| --- | --- | --- | --- |
| 1^st^ author | Ifeanyi | Ngwoke |  |
| 2^nd^ author | Olalekan John | Okesanya |  |
| 3^rd^ author | Nengak Precious | Danladi |  |
| 4^th^ author | Chinemerem Cynthia | Eze |  |
| 5^th^ author | Dada | Oluwasogo Kehinde |  |
| 6^th^ author | Adepeju | Mary Adedire |  |
| 7^th^ author |  |  |  |
| 8^th^ author |  |  |  |
| 9^th^ author |  |  |  |
| 10^th^ author |  |  |  |

Please use an additional sheet if there are more than 10 authors.

**Section 3: Please provide a justification for change. Please use this section to explain your reasons for changing the authorship of your manuscript, e.g. what necessitated the change in authorship? Please refer to the (journal) policy pages for more information about authorship. Please explain why omitted authors were not originally included and/or why authors were removed on the submitted manuscript.**

| We do not make new author additions or removed ay author. We are reordering authorship to reflect the relative contributions made during manuscript development and revision. Some authors provided substantial additional input during the peer review and revision process, which warrants a change in order. Additionally, we are correcting the presentation of names to ensure accuracy. |
| --- |

**Section 4: Proposed new authorship. Please provide your new authorship list in the order you would like it to appear on the manuscript. Please indicate the corresponding author by adding (CA) behind the name. If the Corresponding Author has changed, please indicate the reason under section 3.**

|  | First name(s) | Family name (this name will appear in full on the final publication and will be searchable in various abstract  and indexing databases) | Affiliated institute | E-mail address |
| --- | --- | --- | --- | --- |
| 1^st^ author | Ifeanyi | Ngwoke |  |  |
| 2^nd^ author | Mary Adedire | Adepeju |  |  |
| 3^rd^ author | Nengak Precious | Danladi |  |  |
| 4^th^ author | Chinemerem Cynthia | Eze |  |  |
| 5^th^ author | Oluwasogo Kehinde | Dada |  |  |
| 6^th^ author | Olalekan John | Okesanya |  |  |
| 7^th^ author |  |  |  |  |
| 8^th^ author |  |  |  |  |
| 9^th^ author |  |  |  |  |
| 10^th^ author |  |  |  |  |

Please use an additional sheet if there are more than 10 authors.

**Section 5: Author contribution, Acknowledgement and Disclosures. Please use this section to provide a new disclosure statement and, if appropriate, acknowledge any contributors who have been removed as authors and ensure you state what contribution any new authors made (if applicable per the journal or book (series) policy). Please ensure these are updated in your manuscript - after approval of the change(s) - as our production department will not transfer the information in this form to your manuscript.**

**New acknowledgements:** We do not have new acknowledgements to make.

**New Disclosures (financial and non-financial interests, funding):** We do not have new funding disclosures.

**New Author Contributions statement (if applicable per the journal policy):** Not applicable.

State ‘Not applicable’ if there are no new authors.

**Section 6: Declaration of agreement. *All* authors, unchanged, new and removed *must* sign this declaration.**

(NB: Please print the form, (docu)-sign and return/upload a scanned copy. Please note that signatures that have been inserted as an image file are acceptable as long as it is handwritten. Typed names in the signature box are unacceptable.) *** Please delete as appropriate. Delete all of the bold if you were on the original authorship list and are remaining as an author.**

|  | First name | Family name |  | Signature | Date |
| --- | --- | --- | --- | --- | --- |
| 1^st^ author | Ifeanyi | Ngwoke | I agree to the proposed new authorship shown in section 4 /**and the addition/removal*****of my name to the authorship list** /and the proposed  change in corresponding author | 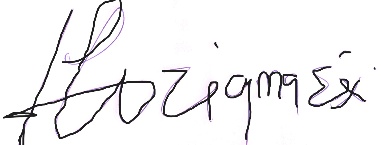 | 02/03/2026 |
| 2^nd^ author | Adepeju Mary | Adedire | I agree to the proposed new authorship shown in section 4 /**and the addition/removal*****of my name to the authorship list** /and the proposed  change in corresponding author | 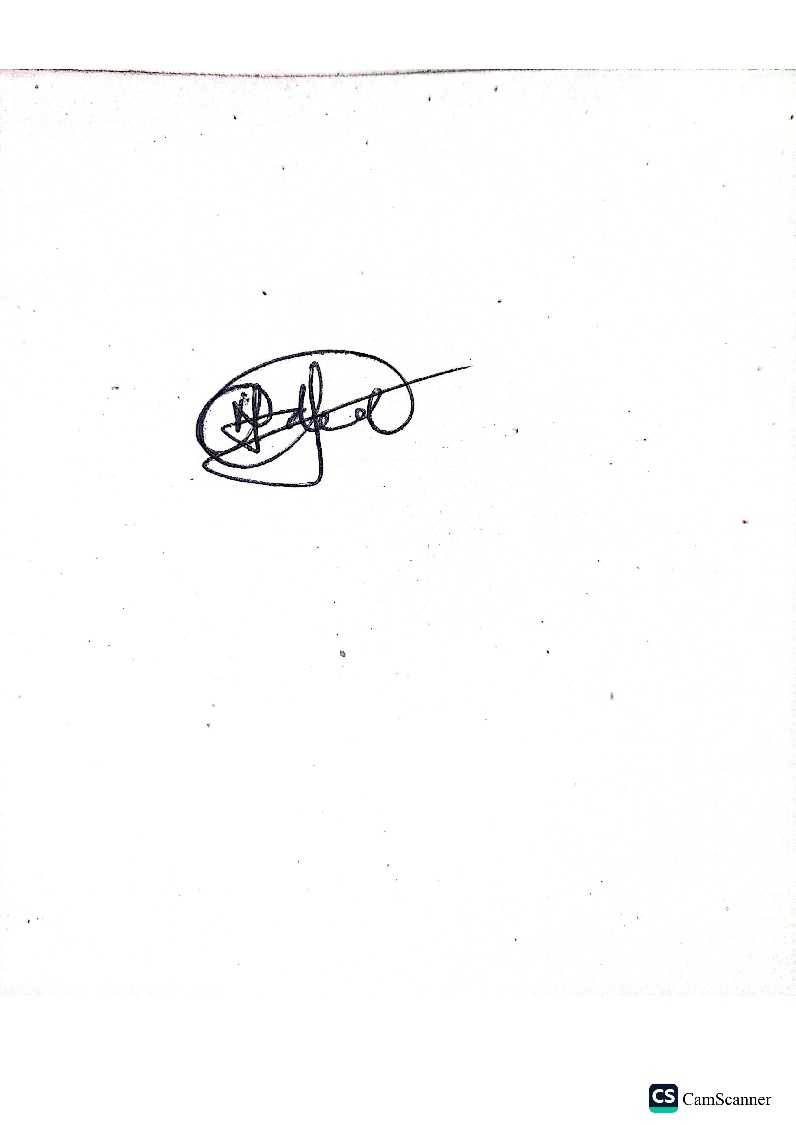 | 02/03/2026 |
| 3^rd^ author | Nengak Precious | Danladi | I agree to the proposed new authorship shown in section 4 /**and the addition/removal*****of my name to the authorship list** /and the proposed  change in corresponding author | 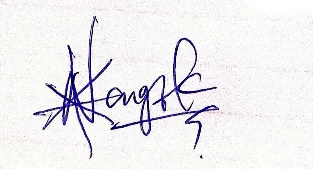 | 02/03/2026 |
| 4^th^ authors | Chinemerem Cynthia | Eze | I agree to the proposed new authorship shown in section 4 /**and the addition/removal*****of my**  **name to the authorship list** /and the proposed change in corresponding author | 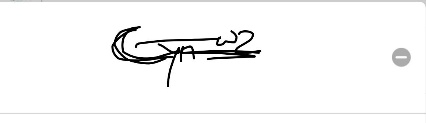 | 02/03/2026 |
| 5^th^ author | Oluwasogo Kehinde | Dada | I agree to the proposed new authorship shown in section 4 /**and the addition/removal*****of my name to the authorship list** /and the proposed  change in corresponding author | 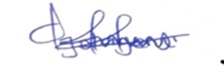 | 02/03/2026 |
| 6^th^ author | Olalekan John | Okesanya | I agree to the proposed new authorship shown in section 4 /**and the addition/removal*****of my name to the authorship list** /and the proposed  change in corresponding author | 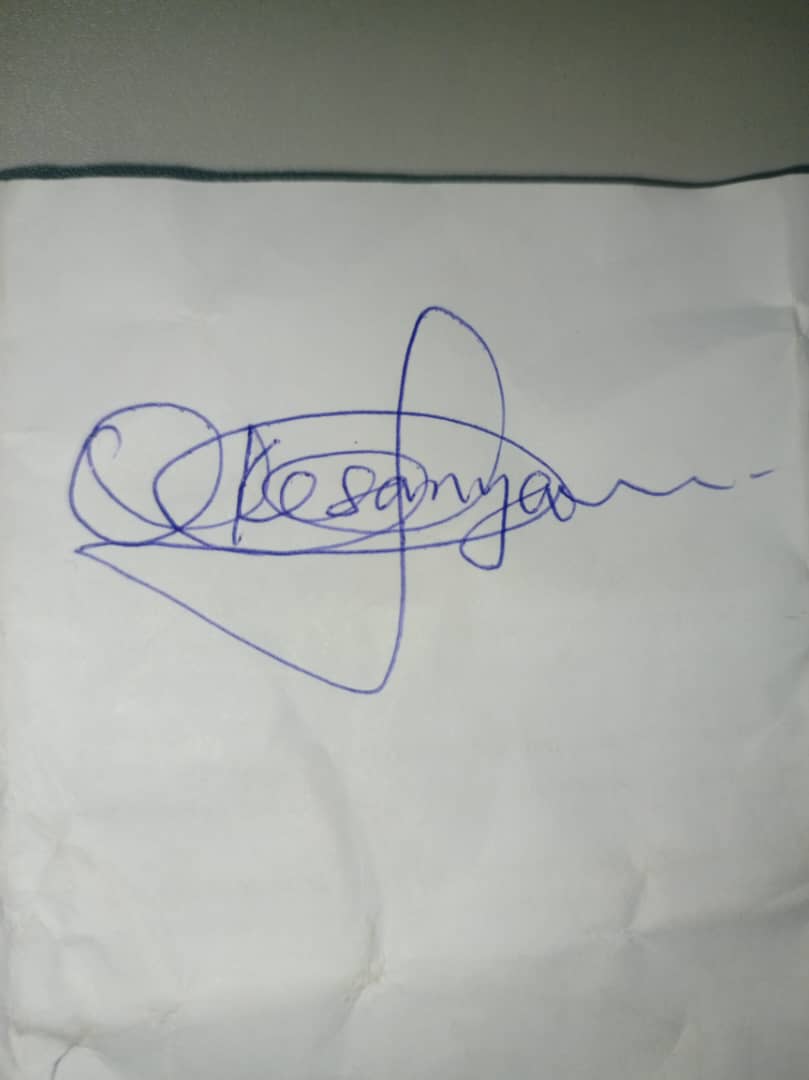 | 02/03/2026 |
| 7^th^ author |  |  | I agree to the proposed new authorship shown in section 4 /**and the addition/removal*****of my name to the authorship list** /and the proposed  change in corresponding author |  |  |

|  | First name | Family name |  | Signature | Date |
| --- | --- | --- | --- | --- | --- |
| 8^th^ author |  |  | I agree to the proposed new authorship shown in section 4 /**and the addition/removal*****of my name to the authorship list** /and the proposed  change in corresponding author |  |  |
| 9^th^ author |  |  | I agree to the proposed new authorship shown in section 4 /**and the addition/removal*****of my name to the authorship list** /and the proposed  change in corresponding author |  |  |
| 10^th^ author |  |  | I agree to the proposed new authorship shown in section 4 /**and the addition/removal*****of my name to the authorship list** /and the proposed  change in corresponding author |  |  |

Please use an additional sheet if there are more than 10 authors.

**In case of author collaborations with formal agreement:**

|  | Name of  consortium/consortia | First name | Family name |  | Signature | Date |
| --- | --- | --- | --- | --- | --- | --- |
| Representative/legal guarantor |  |  |  | I agree to the proposed new authorship shown in section 4 /**and the addition/removal*****of my name to the authorship list** /and the proposed change in  corresponding author |  |  |

**Both added/removed authors should complete the information in the first table under Section 6.**

---- End of form ----
